# What type of social media posts about sustainable construction is better for audience engagement?

**DOI:** 10.12688/openreseurope.17079.2

**Published:** 2024-06-13

**Authors:** Lea Primožič, Franz Dolezal, Rok Prislan, Andreja Kutnar

**Affiliations:** 1 InnoRenew CoE, Koper, Slovenia; 2 University of Primorska, Koper, Slovenia; 3 IBO – Austrian Institute for Building and Ecology, Vienna, Austria

**Keywords:** sustainable construction, communication, social media, engagement, research project outreach.

## Abstract

**Background:**

In an effort to move to a sustainable society, new concepts and findings related to sustainable construction are being developed. With ambition to transfer newly developed knowledge to society, various communication paths are being used. In this study we investigated what kind of messages shared on institutional social media channels (Facebook, Twitter (now renamed to X), and LinkedIn) about sustainable construction create more audience engagement.

**Methods:**

The study consisted of two phases of weekly social media posts. In each phase, 15 posts were published on the same day and time, while engagement was monitored. Three different types of posts were created, that were sequential cycling each week. Type 1 was written informative content related to research activities; type 2 was image content related to the research activities and equipment, with a short text caption of the image; and type 3 was image content with people – scientists working on research activities with a short text caption of the image.

**Results:**

Poisson regression analysis revealed that type 3 posts result in the most audience engagement on LinkedIn, suggesting that using images of people in combination with short text captions is the most effective way to engage social media audiences. These findings can help organizations to use social media to promote sustainable construction and other sustainability-related research. The engagement was lower on Facebook and Twitter (X).

**Conclusions:**

As the science is aiming to be closer to the society, these findings deliver an important insight of science communication through the social media. Although the study delivered several lessons learnt related to science communication through social media studies, it provides an important bases for further studies. Conclusions can support research organizations in improving their science communication.

## Introduction

The field of communication witnessed many changes over time. Many communication models exist that define communication and describe its various elements ( Foulger, 2004). The communication models developed in the past have been adapted also to the field of science with the goal of efficient science communication ( Burns *et al*., 2003). Communication and dissemination of research findings is common practice among scientists. Often, this is also a requirement from research funding bodies. We can define it as a planned process that carefully considers the intended audience and the contexts in which research findings will be presented, with the goal of effectively communicate and engage with the target audience through various interactions ( Wilson *et al*., 2010).

There are several ways to disseminate and communicate about research. Science communication is about sharing findings, increasing public appreciation, increasing knowledge, and understanding, influencing opinions, policy preferences and behaviours, and is about promoting diverse science perspectives for comprehensive societal challenges ( Medicine *et al*., 2017). As the scientific topics related to environmental issues are complex and hard to understand for the general public ( Moser, 2010), the models of communication must be adopted and include not only the transfer of information from the messenger to the audience through a channel, but they must recognize also the context and the meaning of the message ( Jasanoff *et al*., 2001). Improving the population’s beliefs about science and generating social acceptance are also among the aims of science communication. Just like in the communication literature, also in science communication we find one-way communication when scientist share their knowledge to the public, and a dialogue model, when the public, experts and other stakeholders are engaged in a scientific discussion ( Kappel & Holmen, 2019). Social networks are one of the channels offering the possibility of dialog and they became a crucial part of communication strategies.

Social media play an important role in science communication. The emergence of different social media channels has revolutionized the manner in which the general public interacts with scientists and scientific institutions. Social media means a two-way technology-facilitated dialogue, that is more interactive, compared to traditional media ( Kaplan & Haenlein, 2010). It allows information to spread rapidly in a digital community ( Darling *et al*., 2013). Users engage on social media channels with liking, commenting, and sharing posts ( Hwong *et al*., 2017). Scientist are using social media to share their scientific results and the general public is using them to search for information ( Van Noorden, 2014). Scientists are quickly adopting social media for disseminating their research results because science communication is becoming more and more important ( Parsons *et al*., 2014). Science relies heavily on social interaction, and scientists require effective communication and creativity, since inability to convince fellow experts of the significance of one's work can result in being disregarded and irrelevant ( Insall, 2023).

Many social media channels exist, like Twitter (X), Facebook, YouTube, Pinterest, Instagram, and different blogs. Three channels dominate science communication, namely Twitter (X), Facebook, and LinkedIn ( Collins *et al*., 2016). The surveyed scientists use Facebook for personal use and they share scientific results with colleagues, family and friends, and only few believed that it is an appropriate channel to share research with the general public ( Collins *et al*., 2016). Facebook is a social network where users share more personal information ( Skeels & Grudin, 2009), and compared to LinkedIn and Twitter (X), it has the highest number of users – over 2 billion monthly active users ( Dixon, 2023b). Facebook is where users want to be connected with friends, their work and spend time with daily activities such as playing games or being part of groups with similar interests ( Mazman & Usluel, 2010). On the other hand, LinkedIn is dedicated to professional networks ( Skeels & Grudin, 2009). It has over 774 million users and it is the most effective platform in terms of audience engagement ( Dixon, 2022). LinkedIn is known as a professional platform for self-promotion and job recruitment ( Bridgstock, 2019). Twitter (X) is the channel most used by scientists to communicate with other scientists ( Collins *et al*., 2016). Users are connecting with their family, friends and colleagues by sharing short news and status updates ( Bonsón *et al*., 2019). In comparison to Facebook and LinkedIn, Twitter (X) has the lowest number of users – over 350 million users ( Dixon, 2023a). Additionally, it is critical to consider the platform's unique history, which includes political (particularly in the United States) and ownership changes. These factors are likely to have influenced Twitter (X) users' demographics, the type of content they share, and their level of engagement on the platform ( Rohlinger *et al*., 2023). Twitter (X) is very important in terms of policy and journalism ( Lupton, 2014).

In order to be successful in sharing messages on different social media channels there are some factors to consider. For example, it is important to know what is the best time of day to post for reaching the widest audience. However, it is hard to predict which time and day is the best to post on different social media channels. Nevertheless, there is data that suggest peak times. Peak days and times differ for Facebook, Twitter (X), and LinkedIn. Tuesday from 9am to 12pm (in all time zones) was identified as a peak period in all three channels ( Post, 2021). This data can be changing quickly as different social media algorithms are changing constantly. The length of social media posts is another characteristic that might influence the audience engagement. Some authors say that the length of posts does not affect engagement ( Li & Xie, 2020), still several web blogs ( Geyser, 2022; McLachlan, 2022) report that 80 characters is an average post length effective on social media.

On social media, informative and emotional type of messages tend to create more engagement ( Li & Xie, 2020). Adding visual elements help create more engagement ( Bruce *et al*., 2017; Goldfarb & Tucker, 2011; Li & Xie, 2020; Pieters & Wedel, 2004). Images of people in print ads increase their effectiveness ( Li & Xie, 2020). Top features for engaging messages on Facebook and Twitter (X) in space science are photos and other visual elements ( Hwong *et al*., 2017). Visual communication is more persuasive, it gets more attention and enhances memorability of a message. Images also induce positive emotions which influences our persuasion towards a topic ( Escalas, 2004). In a meta-analysis study they found out that adding a realistic photograph to a text message increases the persuasion ( Seo, 2020). Messages must be clear and simple, and they need to fill in the knowledge gaps between the communicator and the audience ( Moser, 2010). Additionally, messages need to get audience attention and must have an important emotional impact ( Korhonen *et al*., 2016; Moser, 2010). Another important implication of social media communication is the lifespan of social media posts that is relatively short, and it is different for each social media channel. The lifespan of Twitter (X) posts is 15 minutes, Facebook 6 hours, and LinkedIn 30 hours ( Ripo, 2021). Using hashtags in social media posts is an integral part of communication. It started as an idea to use the hashtag in front of the keyword allowing users to link and easily find similar content. Later we witnessed how it helped in creating several social movements (Me too, black lives matter, …) or other marketing campaigns. Popular press recommends to use between two and three hashtags ( Lee, 2016; Schwab, 2021; Socialbakers, 2021).

Social media and appropriate marketing and communication strategies can make a big difference also in consumer behaviour that impacts the climate change, and the needed sustainability transformation of our society. Research organizations, and companies are using social media when talking about sustainability related issues to the general public ( Clark, 2008). As consumers gain interest in environmental topics it causes companies to embrace greener ways of operating and, in return, communicating about this. Consumers are searching on social media for products that are more sustainable. Therefore, using social media became an important tool for communication. Global consumer brands are emphasizing environmental aspects in their online sustainability communication ( Primožič & Kutnar, 2022). Green advertising on social media is an important marketing tool companies should use. Efficient communication about sustainability related topics, among which also sustainable construction, should be based on a clear communication strategy ( Dawkins, 2004). A recent study suggests that messages about environmental and social issues, shared on Twitter (X), engage stakeholders the most (as compared to other sustainable development goals), while the relationship between the stakeholder engagement and image or video type of message was not supported ( De Luca *et al*., 2022).

In our study, communicating about sustainable construction related topics was approached by communicating on social media about research activities of a project J4-3087 Engineered wood composites with enhanced impact sound insulation performance to improve human well being financed by Slovenian Research and Innovation Agency and Austrian Science Fund. The aim of the project is to address climate change issues and the negative impacts of the building industry. With research projects dedicated to developing solutions in different fields that contribute to lowering the effects of climate change, significant progress can be achieved. The most promising way to limit the negative impacts of the building industry for the environment is to promote the use of renewable materials. There are several challenges that must be addressed if we want to promote the wider use of renewable materials in buildings. In this perspective, the most common prejudice is that wooden buildings suffer from low sound insulation. More specifically, it is a challenge to achieve high impact noise insulation because the structural elements are light, more sensitive to excitation, and generally lack sufficient damping that would restrain the propagation of vibrations ( Bard Hagberg, 2018). This engineering issue is addressed in this research project. Researchers are investigating the potential of creating wooden composites based resilient layer to limit impact noise in buildings. The main purpose of the project is to improve the impact noise insulation in wooden buildings by engineering a sustainable resilient layer. This is done by a fundamental assessment of the annoyance of impact noise in the built environment that surmounts standardized insulation ratings. InnoRenew CoE is the leading partner. Other project partners are University of Primorska, University of Ljubljana, IBO Austrian Institute for Building and Ecology, and TU Vienna University of Technology.

This study was conducted in the framework of the above-mentioned research project. We investigated what kinds of messages related to this project we need to communicate on different social media channels to create more audience engagement. The overall objective of this research is to study the type of messages that should be shared on institutional social media channels (Facebook, Twitter (X), and LinkedIn) about sustainable construction related topics in order to create more engagement of the audience.

In order to follow the main objective of this study, we formulated the following research questions (RQ):

RQ1: What are the effective types of messages to be shared on social media channels (Facebook, Twitter (X), and LinkedIn) to increase engagement of the target audience towards sustainable construction solutions?

RQ2: Are there any differences among the three social media channels used in terms of message effectiveness?

RQ3: Are there any differences connected with time/season?

RQ4: Are there any differences connected with the added alternative text to social media posts with an image?

## Materials and methods

For this study the content and materials were prepared and collected in English and posted on institutional social media channels of the research institute InnoRenew CoE. InnoRenew CoE is a research institute dedicated to research and innovation in the field of renewable materials and healthy built environments. Two key research areas are wood modification and restorative environmental and ergonomic design (REED), which supports creating positive health impacts for building users and the environment.

Based on data obtained on April 17, 2023, InnoRenew CoE is active on Facebook with 1953 followers, Twitter (X) with 1430 followers and LinkedIn with 2571 followers. The institute is posting only content in English on its social media channels. Compared to other, similar, research institutions from Slovenia, for example the Slovenian National Building and Civil Engineering Institute, InnoRenew CoE has higher number of followers, while in comparison with bigger international research institutes, like the RISE Research Institutes of Sweden, its numbers are lower.

The audience of InnoRenew CoE’s social media channels on Facebook is mostly in the age group from 35–44, and 53% of the audience is female. Facebook followers are mostly from Slovenia, followed by USA, Italy, and Germany. The LinkedIn audience is also mostly from Slovenia, followed by Turkey, Finland, and Croatia. Follower’s job function is predominately from the research field, followed by education, business development, engineering, and operations. The industry that is covered from LinkedIn audience is the higher education, research, paper and forest products manufacturing, civil engineering, and construction. Unfortunately, Twitter (X) analytics no longer provides audience insights, however according to a previous study from the United States, the audience is mostly younger people who are more likely to be highly educated and have higher incomes ( Wojcik & Hughes, 2019).

The shared content on social media channels was highly interdisciplinary, connecting different fields with the same goal – to advance more sustainable solutions in the built environment. Each of the project partners were preparing content related to their research project tasks. The InnoRenew CoE was communicating about building and testing the wood-based resilient layer for use in floating floors, about setting up the vibration exposure device, and the Ambisonics loudspeaker system for tests in the anechoic chamber. Most topics were connected to the field of wood composites, impact noise and the dynamic characterization of structures. The IBO Austrian Institute for Building and Ecology was preparing content related to impact noise levels, sound measurements, as well as different types and acoustic properties of wooden component, and the impact and the descriptors of different material types and layers. Therefore, social media posts were related to human well-being, acoustics, and impact noise. The TU Vienna University of Technology shared content about acoustics numerical modelling. In the shared content, scientists are presenting a Finite Element Method (FEM) model as a visual representation of a floor structure. Furthermore, the FEM was used to predict the influence of floor structures on the impact sound insulation of wooden ceilings. Additionally, results of the FEM Model are modelling the dynamic force excitation of the floor structure by a tapping machine and the necessary measurement setup for validation of the model. The University of Primorska prepared content about human well-being related to indoor materials and noise. Posts were about measuring stress in people, stress caused by exposure to noise, and how different materials used indoors can mitigate noise disturbance. University of Ljubljana prepared content related to mechanical models and the related boundary conditions that well describe the elastic response of the developed resilient layer.

The study was conducted in 2022 and consisted of two phases. The first phase took place from May 3 until August 9, and the second phase took pace from August 30, until December 6. In each phase 15 posts were published, one every Tuesday at 10 am on InnoRenew CoE’s social media channels (Facebook, Twitter (X), and LinkedIn). On each platform the same post was published. Monitoring of engagement ( Table 1) took place every Thursday at 10 am. We collected different information related to the effectiveness of communication for the studied social media channels, since not all include the same parameters. However, we clarified in advance what each parameter means in order to properly compare the data. We monitored engagement on Twitter (X) and LinkedIn, which is measuring the total number of times a user has interacted with a message. We compared it with the number of clicks on the post on Facebook. Engagement is an important metric revealing that the content is compelling, and it leads to discussion. Further, we measured impressions on Twitter (X) and LinkedIn, which are reflecting the number of times a message was seen, hence we can compare it with the reach of a message on Facebook. Impressions are important to understand if the message is reaching a vast audience. Therefore, in the analysis we used the term engagement which includes engagement on Twitter (X) and LinkedIn and number of clicks on Facebook, and the term impressions which includes impressions on Twitter (X) and LinkedIn, and reach on Facebook. Additionally, we collected data about number of likes, comments, and shares received on each social media channel.

**Table 1.** Quantitative measures monitored on social media.

	Facebook	Twitter	LinkedIn
Engagement		X	x
Number of clicks	x		
Reach	x		
Impressions		X	x
Number of likes, comments, and shares	x	X	x

**Figure 1.**
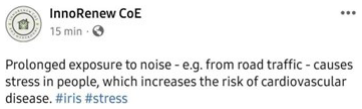
Example of Type 1 post on Facebook.

Three different types of posts were created. Type 1 posts ( Figure 1) was written informative content (80–150 characters with spaces). Content was related to the project research activities and research fields. All content was of informative nature.

**Figure 2.**
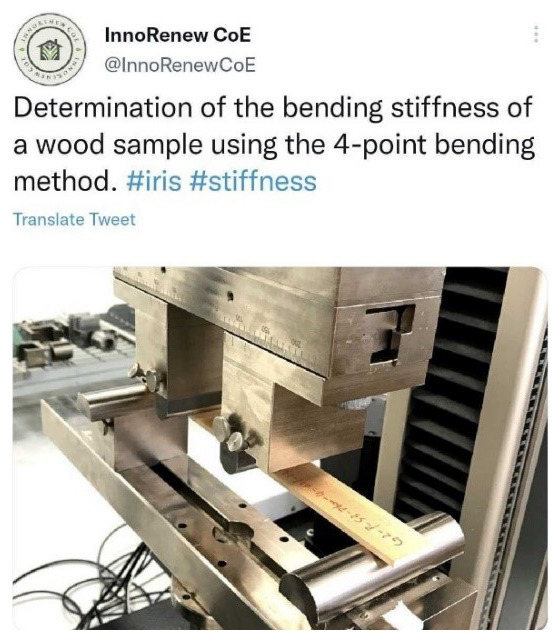
Example of Type 2 post on Twitter (X).

Type 2 posts ( Figure 2) was image content related to the project research activities and research equipment used in the project together with short text caption of the image.

**Figure 3.**
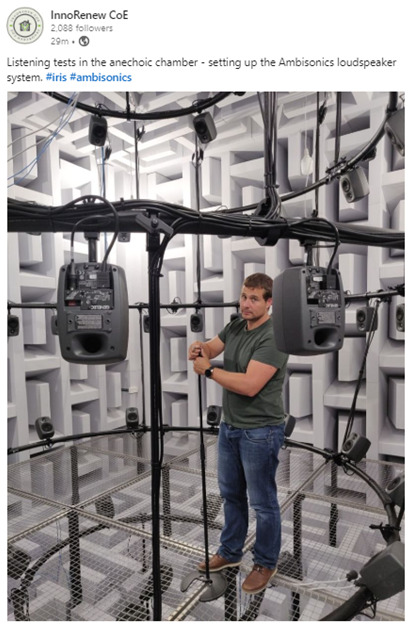
Example of Type 3 post on LinkedIn.

Type 3 posts ( Figure 3) was image content with people – scientists working with research equipment used in the project together with short text caption of the image.

Each post contained two hashtags – one was related to the project name and always the same (#iris), while the other differed depending on the content.

Overall, this study included 30 posts related to wood composites, sound insulation, building acoustics, human well-being, and modelling of the floating floor. In Table 2 we present the number of the 30 social media posts, their type and the respective field they were covering.

**Table 2.** Number of analysed posts per research topic and type of post.

	Type 1	Type 2	Type 3	Total
Wood composites	3	2	1	6
Impact noise	1	2	4	7
Acoustics	2	3	2	7
Human well-being	3	2	3	8
Modelling	2	1	2	5
				33 *

* In total there were three social media posts (one of type 1 and two of type 3) that were covering two different fields and not just one, this is why in the Table 2 the total number is 33.

Additionally, we posted four additional weekly posts (at the same time and day each week) of only type 3 on social media, with the same content as we posted in previous two phases of type 1 and type 2. We aimed to verify that the studied differences in engagement can be attributed to different types and not the content.

## Results

The analysis performed has delivered important findings to be used in future science communication through social media. We are providing the results of the study and in the discussion point out the need for future research in the field.

The analysis of social media posts included performance of 30 social media posts, 15 from phase 1 and 15 from phase 2. All social media posts were somehow evenly distributed regarding the content; however, we can notice a small difference. The topic of human well-being was the one mostly communicated about, followed by social media posts about impact noise and acoustics, wood composites, and modelling ( Table 2). For the analysis we applied Poisson regression as our data was count data of relatively low values and we had a small sample size (30 posts). Poisson regression is an analysis known as a generalized linear model. It is used for modelling data predictions. The outcome variable is a count of the number of events that occur in a fixed period of time ( Coxe *et al*., 2009). The data obtained from our study fit these requirements.

Since we analysed three different social media channels, we have to note that although there may be some overlap of individuals that follow the social media accounts on each platform, we have treated the audience in each platform as independent from one another.

**Figure 4.**
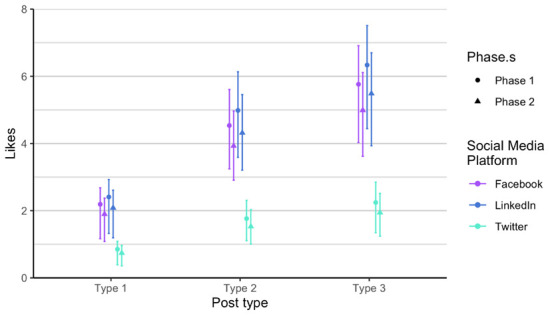
Number of likes for each post type on Facebook, Twitter (X), and LinkedIn.

**Figure 5.**
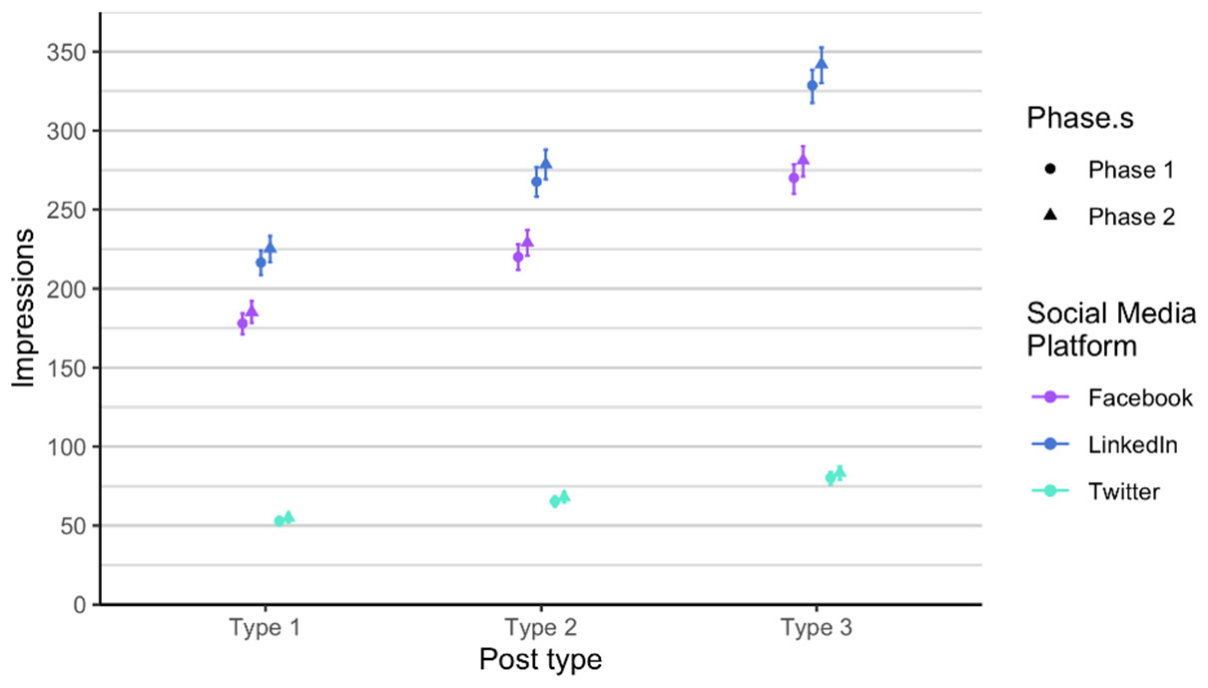
Impressions for each post type on Facebook, Twitter (X) and LinkedIn.

**Figure 6.**
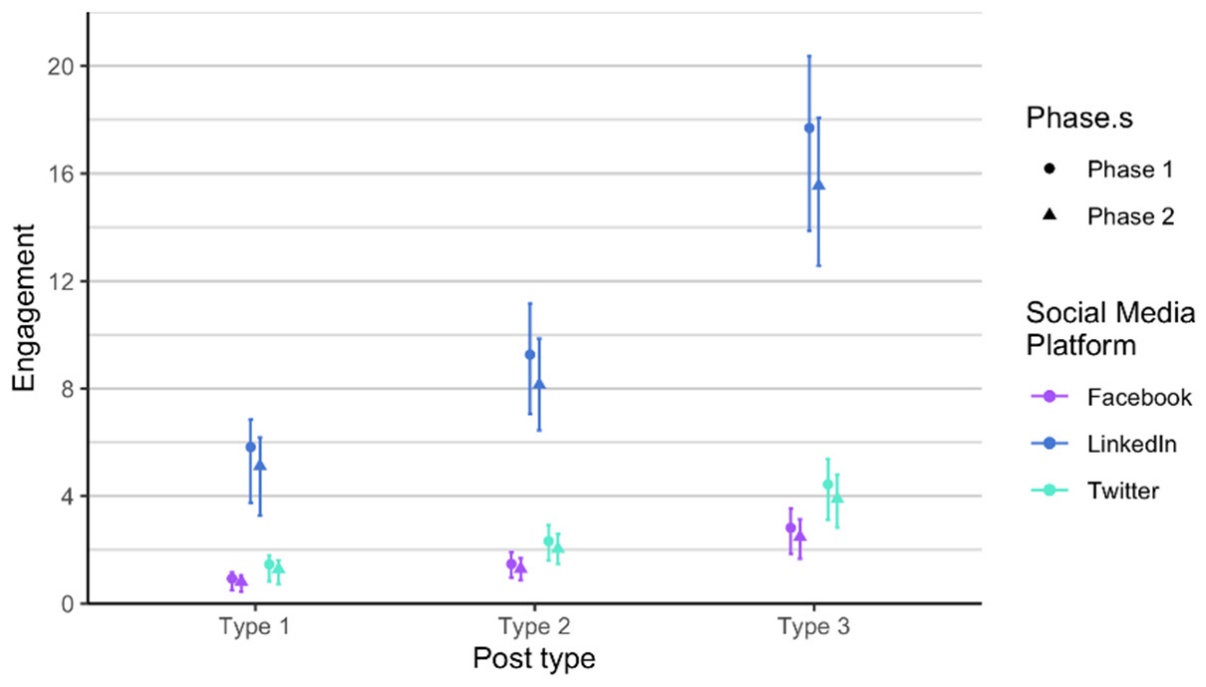
Engagement for each post type on Facebook, Twitter (X) and LinkedIn.

As related to the RQ 3 and RQ4, we first analysed each phase of posts separately, in order to verify if there is any difference between the two phases, considering the time dimension (season) and the added alternative text. The alternative text was added to posts in phase 2. It means that each social media post that contained an image had also the written alternative text that is explaining what is on the image, enabling wider accessibility. It is usually used to describe elements, like photos and other types of data visualizations. The difference between the two phases is not statistically significant when comparing number of likes, impressions and engagement. Number of comments and shares was very low, so we did not include these two parameters in the analysis. Furthermore, number of likes on Twitter (X) is always lower compared to number of likes on LinkedIn and on Facebook, in all types of posts. On the other hand, number of likes on LinkedIn and Facebook is relatively similar in all types of posts and both phases ( Figure 4). We observed the same result in terms of impressions on Twitter (X) as it is always considerably lower ( Figure 5). Impressions are higher on LinkedIn compared to Facebook, regardless of type and phase. Engagement is the lowest on Facebook ( Figure 6), a bit higher on Twitter (X), and the highest on LinkedIn, regardless of post type.

As there was no significant difference between the phase 1 and phase 2, the overall analysis in relation to RQ1 and RQ2, included all 30 social media posts, regardless of phase, was performed.

We compared the number of likes on all three social media channels, the engagement on Twitter (X) and LinkedIn with number of clicks on Facebook, and the reach on Facebook with impressions on Twitter (X) and LinkedIn. As stated above, we compared parameters that are differently named on the social media channels but according to the definitions they measure the same thing.

**Figure 7.**
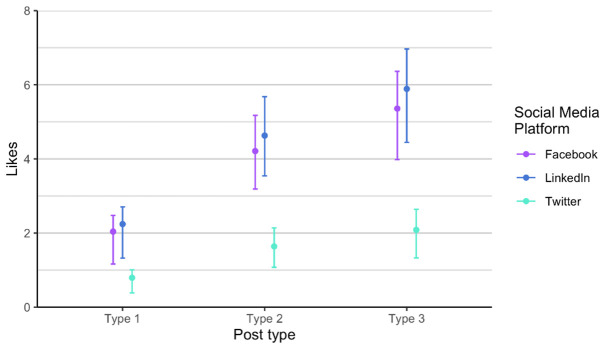
Number of likes for each type for Facebook, Twitter (X) and LinkedIn (phase 1 and phase 2 combined).

With the Poisson mixed model, we modelled the data predictions based on the number of likes ( Figure 7). We found out that each type is different and that the most likes are of type 3 posts, across all social media channels. Effect of type is consistent. We expect that on Facebook and LinkedIn, the number of likes is approximately the same, regardless of type, and we expect number of likes on Twitter (X) to be considerably lower. The number of followers in the Twitter (X) account is the lowest therefore this result is at least somehow expected. Additionally, Twitter (X) is the social media with the lowest number of users in general. On average the higher number of likes is on LinkedIn for all posts types.

**Figure 8.**
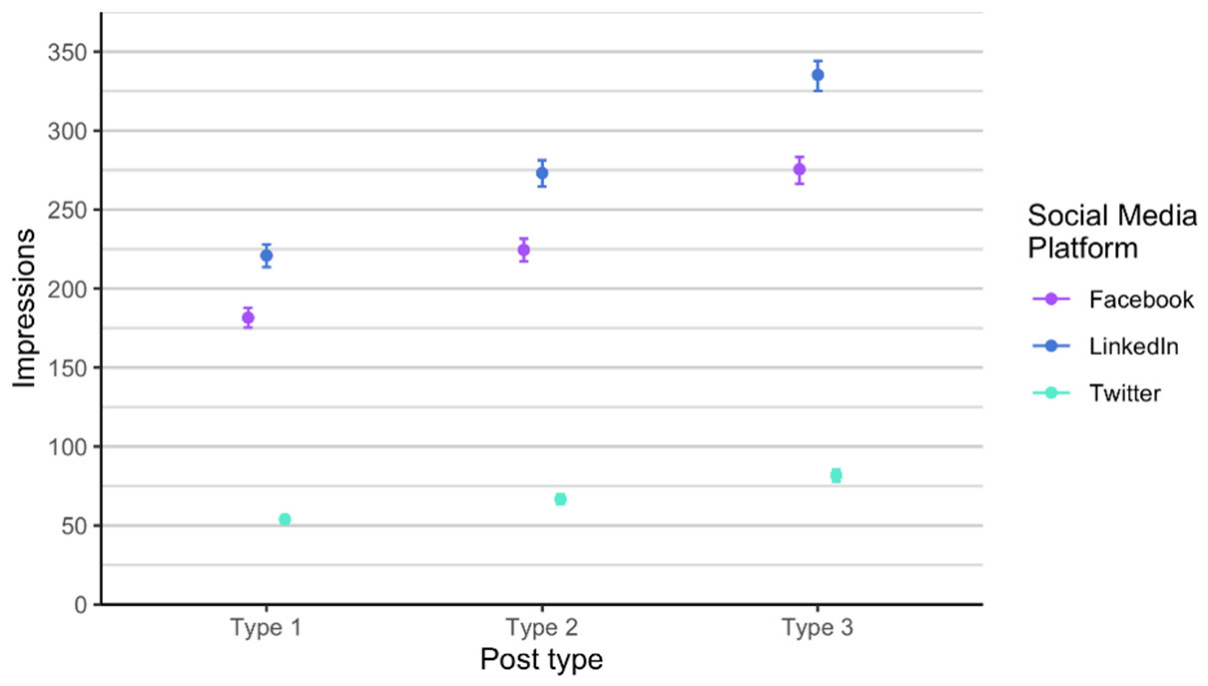
Impressions for each post type on Facebook, Twitter (X) and LinkedIn (phase 1 and phase 2 combined).

Similarly, the type 3 posts shared on LinkedIn were highest in impressions ( Figure 8). There is less variance in posts on all channels compared to the number of likes ( Figure 7). Type 3 posts performed better regardless of the social media channel.

**Figure 9.**
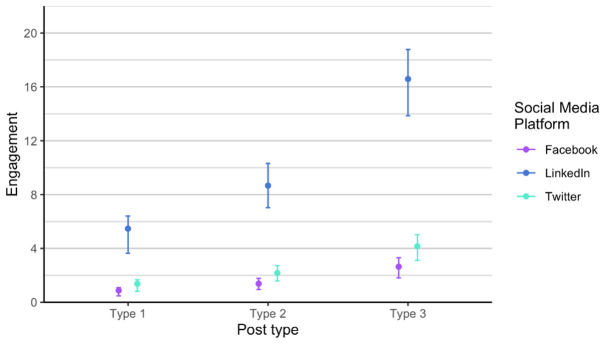
Engagement for each post type on Facebook, Twitter (X) and LinkedIn (phase 1 and phase 2 combined).

In regard to engagement, posts performed slightly different. The highest was still on LinkedIn, and type 3 posts performed the best for all channels, while lowest on Facebook, which is not the case for number of likes and impressions ( Figure 9). We discovered that Twitter (X) and Facebook are fairly consistent in terms of engagement, while we notice a lot of variability on LinkedIn. The reasons for the variance can be found in the changing algorithms on social media, different preferences of users, and other happening on the day of the post. For example, the social media post shared on LinkedIn with lower engagement were shared on dates when there was several news stories related to the current war or Brexit developments.

Further, we found that there was only one comment on LinkedIn for type 3 post, and there were only five shares of posts, of which one on Twitter (X) for type 3, and three on Facebook for type 1 and one on Facebook for type 3. These findings indicate that users may not be comfortable commenting on topics connected to sustainable construction, or that they are not interested in engaging in conversations on social media to develop new relationships. This was suggested also by previous research which discovered that certain users are afraid to engage in social interactions online ( Ellison *et al*., 2014). Sharing a post is even a more visible action than commenting or liking, but it is also less direct, as found in past studies ( Li & Xie, 2020).

Finally, with the analysis of the additional weekly posts of only type 3 we confirmed that the studied differences of engagement can be attributes to the studied factors. Namely, we discovered that if we share the same content in as different type, the result is the same (e.g. the shared content does not affect our results). On average, these additionally shared posts of type 3 performed better in terms of engagement, compared to the same content shared as type 1 and type 2.

## Discussion

In our study we found that social media posts related to sustainable construction topics, shared on social media channels of a research institute, containing an image with people, performed the best on different social media channels. This is why, in relation to the first research question we discovered that social media posts with an image of people (type 3 post) generated highest engagement, impressions and likes. Literature suggests that visual elements such as images are helpful in terms of creating engagement ( Bruce *et al*., 2017; Goldfarb & Tucker, 2011; Hwong *et al*., 2017; Li & Xie, 2020; Pieters & Wedel, 2004). However, there are also some contradictory findings where the relation between the image in engagement was not confirmed ( De Luca *et al*., 2022). Several scholars studied the persuasion and engagement of visual elements in advertising and marketing areas, but to our knowledge there is a lack of studies in this field related to research of sustainable construction. Therefore, we uncovered a novel insight regarding social media posts of research organizations. The reason why people engage with some posts on social media, and some not, is hard to predict and can be affected by several factors. Some authors ( Chung & Darke, 2006) suggest that people who are engaging more often have certain personal characteristics, like the urge to provide useful information and to influence how other people perceive them. The content of the message and how it is said also influences users and engagement in online discussion. Emotional, social, cognitive and descriptive words help to reach greater engagement ( Moore & McFerran, 2017).

With reference to the second research question, LinkedIn performed the best in terms of message effectiveness. This is somehow expected given that the account from where the social media posts were shared had the highest number of followers, compared to the other two channels included in the study. Additionally, in comparison to Facebook, LinkedIn has less users overall, however it is the platform known to have an engaged audience ( Dixon, 2022). Moreover, we found that in terms of likes, all types of social media posts performed similarly on Facebook and LinkedIn, while on Twitter (X) the number is considerably lower. Twitter (X) is the channel that is more dedicated to political discussions ( Lupton, 2014) this is why users might not have a high interest in research topics. In contrast ( Collins *et al*., 2016) argues that scientists are using Twitter (X) as a means to communicate with their peers. However, the latest changes in ownerships, could potentially have impacted the communication dynamics. The institutional account from where posts were shared has the least number of followers on Twitter (X), compared to the other two social media channels. As previously stated, LinkedIn is a professional networking channel, therefore users are focused on their profession and career development, which leads to more meaningful interactions. Similarly, content uploaded on LinkedIn is more likely to increase user engagement as users find it more useful and relevant to their professional development. In comparison, Twitter (X) and Facebook typically feature content of a different sort, and people do not engage as much because they utilize them for other goals other than professional.

Regarding the third and fourth research questions we found that the time dimension (season) and alternative text does not influence the engagement and other monitored parameters, as the analysis revealed there was no significant difference between posts of phase 1 and phase 2. Due to social media algorithms constantly changing, it is difficult to forecast when is the optimum time to post ( Post, 2021). The uncontrolled changes of social media algorithms, which the account holders cannot influence, can also affect the reason why there is no significant difference between the two phases in relation to adding alternative text.

Furthermore, we identified limitations of our study. First, the audience in this study is related to one institution (research organization). Accordingly, the audience may have its own characteristics which are not representative of the larger population, potentially limiting the generalizability of the findings ( Berkowitz & donnerstein, 1982). Knowing the exact characteristics of our audience and understanding the potential for audience overlap and relapse is a significant limitation. With available analytical tools the audience was studied on each social media channels, however these functions are limited and not all social media channels offer the same kind of data. Comparability of the three social media channels is an additional limitation ( Kietzmann *et al*., 2011). The number of followers is different on the three channels and there might be an overlap between followers. However, in the analysis we did account for repeated measures of the same post on different platform by including the unique post ID as a source of random error. While it is likely there is some duplication of individuals between platforms, we do not have the information to determine if the same individuals responded to the posts on multiple platforms. Despite these limitations we believe the data provides valuable insights into the performance of the posts between platforms, as our study revealed that regardless of the social media channel, posts containing an image with people, performed the best. We suggest that future studies investigate about the characteristics of audience on different social media channels and its effects on post engagement to address these limitations more comprehensively.

Moreover, the fact that posts were shared from an institutional account and not a physical person can influence the results. Appropriately interpreting findings is difficult because it is challenging to determine an accurate explanation for discrepancies and variances in social media posts. This is connected also to the fact that the algorithm on social media channels are constantly changing ( Post, 2021). On top of that, we must stress that the content for social media posts was prepared in collaboration with researchers from different research organizations. This might have influenced the results as the language used was more understandable for professional and scientific stakeholders, rather than the general public reached on some social media channels. If the content would be prepared by communication practitioners or social media specialists, perhaps the findings would be different (Miller - Carpenter & Lertpratchya, 2016). At the same time, this is an important implication for further studies which should focus on expanding the knowledge in this area.

We are aware that such social media studies are very complex to perform and include many external factors that are influencing the outcomes, such as the variation in follower counts across social media platforms, the credibility of accounts, and the diverse audience characteristics ( Soares *et al*., 2021). However, we believe that studies like this one, are very important as they contribute to a more scientific approach in understanding the dynamics of social media engagement, despite the limitations and complexities involved.

Overall, this finding can serve to help other communication practitioners and researchers to improve their communication and dissemination activities on social media about their research projects, and to more effectively promote their project activities related to sustainable construction. Our results show that posts with an image of a person and short text caption are best in terms of different parameters that are important for reaching the audience.

Future studies should consider finding a way to encourage the audience to engage through commenting or sharing with their own thoughts, and although the language in science is English, communication to target audience out of the scientific and professional community should be performed in local languages.

In the field of sustainable construction we can expect that the New European Bauhaus ( *New European Bauhaus*, n.d.) principles in developing new solutions together, sustainable, and beautiful will significantly influence the science communication in the field. It is attempting to bridge research and practice. With further studies on social media use in science communication we can significantly contribute to these ambitions.

## Conclusion

This study investigated the effectiveness of different types of social media posts, shared on institutional Facebook, Twitter (X), and LinkedIn accounts, about sustainable construction related topics. Findings suggests that social media posts with an image of a person generate the highest engagement. These findings provide an important message for communicators and researchers that want to improve their communication activities on social media and assuring broader outreach of their research projects.

In addition to communication experts and researchers, these findings can also serve policy makers as more communication about sustainable construction is needed, either on social media or on other channels, to promote and enhance its wider use. Encouraging communication about sustainability and sustainable construction related topics is crucial to raise awareness and help to transition to a more sustainable society. Policy makers are already taking important steps in this direction by creating and adopting strategies and initiatives, such as the New European Bauhaus in Europe. However, additional communication efforts are needed to enable the transition to a sustainability-oriented society.

Future research should examine effectiveness of different languages, addressing other relevant stakeholders such as architects, constructors, engineers, and other professionals, and by involving communication experts in the creation of the content. More social media channels (Instagram, Reddit, TikTok etc.) could be added to the sample and apply the presented methodological approach to further investigate and expend findings. In future studies, partners involved in the study could be sharing, liking and reposting social media posts and thus investigate the potential of extended reach. Additionally, future research should place more focus on effects of diverse audiences to post engagement.

Moreover, it is worth noting that the social media posts in this study covered highly interdisciplinary research topics, from acoustics to wood composites and psychology. Potentially, there is a need for a more focused and gradual approach to social media communication about sustainable construction topics. Further research should focus on determining which topics should be prioritized and communicated to wider audience on social media. Accordingly, a more holistic understanding of sustainable construction topics in society can be built. To our knowledge there is a lack of prior studies investigating the engagement of different types of social media messages, about a research topic connected with sustainable construction, therefore we believe such studies can be an important first step in the direction of better understanding the social media communication. The information and metrics monitored and observed in this study can help sustainability communication experts and researchers to create impactful messages and to redefine strategies in order to promote more sustainable construction practice, and raise awareness. Furthermore, it is crucial to learn how to use social media in science communication and contribute to the so much needed transformation of engaging society in science.

## Data Availability

Zenodo: Data for the social media study of sustainable construction communication https://doi.org/10.5281/zenodo.10454501 ( Primožič *et al*., 2024) This project contains the following underlying data: social media study_240108_md_v1.txt socialmediastudy_230103_d_p1_v1.csv socialmediastudy_230103_d_p2_v1.csv socialmediastudy_230103_d_p3_v1.csv Data are available under the terms of the Creative Commons Attribution 4.0 International license (CC-BY 4.0).
