# Supplementary material for: What type of social media posts about sustainable construction is better for audience engagement?
Source: Open Res Eur. 2024 Jun 13;4:24. Originally published 2024 Feb 12. [Version 2] doi: 10.12688/openreseurope.17079.2 (PMC11269976; doi:10.12688/openreseurope.17079.2)
Supplement: Peer review history

# Reviewer report: Reviewer response for version 2

**DOI:** 10.21956/openreseurope.19276.r42014

**Published:** 2024-07-24

**Authors:** Florin Nechita

**Affiliations:** 1 Transilvania University of Brasov, Brasov, Romania

The research is relevant for communicating about sustainable construction in a research project dissemination context. As most research dissemination projects have either no communication budgets or very limited budgets, this study is also relevant from this perspective. The researchers presented their case study by communicating three types of content distributed across three different social media platforms. However, most communication campaigns on social media platforms have budgets for running A/B testing of different messages and testing the effectiveness on each channel. Therefore, the results of this study are limited to research project dissemination in a zero-budget context. It is assumed that there was a zero budget for communication on social media, as this information is not provided. The authors should include as a limitation the fact that testing the video content was not considered in their experiment.

If applicable, is the statistical analysis and its interpretation appropriate?

Yes

Is the study design appropriate and is the work technically sound?

Yes

Is the work clearly and accurately presented and does it engage with the current literature?

Yes

Are the conclusions drawn adequately supported by the results?

Yes

Are sufficient details of methods and analysis provided to allow replication by others?

Yes

Are all the source data and materials underlying the results available?

Yes

Reviewer Expertise:

NA

I confirm that I have read this submission and believe that I have an appropriate level of expertise to confirm that it is of an acceptable scientific standard.

# Reviewer report: Reviewer response for version 1

**DOI:** 10.21956/openreseurope.18455.r38969

**Published:** 2024-05-15

**Authors:** Pipiet Larasatie

**Affiliations:** 1 Arkansas Center for Forest Business, University of Arkansas at Monticello, Monticello, Arkansas, USA

The research idea is innovative, and I appreciate how the authors are trying to do the operationalization with some acknowledged limits. My suggestion is to have different sections between Introduction and Theoretical Background (Literature Review). The current introduction section reads more like a theoretical background. I would suggest having an introduction that gives a non-technical audience a general overview of the topic. This is important as the authors are trying to do interdisciplinary research (e.g., communication, social media, sustainability, and construction).

 I am also curious about how the authors address research questions. In my opinion, the research questions resemble specific and testable hypotheses. If my guess is right, with the same spirit of reaching a non-technical audience, I would suggest breaking down the theoretical background per hypothesis.

 Kudos for your hard work.

If applicable, is the statistical analysis and its interpretation appropriate?

Yes

Is the study design appropriate and is the work technically sound?

Yes

Is the work clearly and accurately presented and does it engage with the current literature?

Yes

Are the conclusions drawn adequately supported by the results?

Yes

Are sufficient details of methods and analysis provided to allow replication by others?

Yes

Are all the source data and materials underlying the results available?

Yes

Reviewer Expertise:

Forest products marketing; forest sector competitiveness; sustainable business management.

I confirm that I have read this submission and believe that I have an appropriate level of expertise to confirm that it is of an acceptable scientific standard.

# Response

**Published:** 2024-05-21

**Authors:** Lea Primožič

**Affiliations:** Not Applicable, Slovenia

*Dear reviewer. Thank you for the suggestions, feedback and the manuscript acceptance for publishing. We appreciate your recognition of the innovative nature of the presented study and your acknowledgment of the challenges associated with it. In our study we were guided by research questions as our research is exploratory and the studied topic is complex. This led us also in the analysis, results and discussion part. However, we will assure to take into consideration all the suggestions in the future similar studies that we aim to perform.*

# Reviewer report: Reviewer response for version 1

**DOI:** 10.21956/openreseurope.18455.r38366

**Published:** 2024-03-06

**Authors:** Esra Kurul

**Affiliations:** 1 School of the Built Environment, Oxford Brookes University, Oxford, England, UK

There is very little engagement with the literature. This is at the beginning of the paper and is mainly concerned about communciation. However, the realities of scientific communication through social media have not been explored. 

 The structural characteristics of the 'networks' of the InnoRenew CoE on these three different channels, have been provided at the beginning of the Materials and methods section. This demographic data has not been used to evaluate the 'measures of engagement' on this different platforms. 

 The discussion is mainly based on the number of impressions, shares and comments. The differences between these 'measures of engagement' by different users has not been established in enough depth. For example, there is a claim that "users may not be comfortable commenting on topics connected to sustainable construction, or that they are not interested in engaging in conversations on social media to develop new relationships." As far as the paper is concerned, this correlation is now based on any evidence.

If applicable, is the statistical analysis and its interpretation appropriate?

Partly

Is the study design appropriate and is the work technically sound?

Partly

Is the work clearly and accurately presented and does it engage with the current literature?

Partly

Are the conclusions drawn adequately supported by the results?

Partly

Are sufficient details of methods and analysis provided to allow replication by others?

Yes

Are all the source data and materials underlying the results available?

Yes

Reviewer Expertise:

Sustainability transitions, built environment, social networks.

I confirm that I have read this submission and believe that I have an appropriate level of expertise to confirm that it is of an acceptable scientific standard, however I have significant reservations, as outlined above.

# Response

**Published:** 2024-05-21

**Authors:** Lea Primožič

**Affiliations:** Not Applicable, Slovenia

*The authors are appreciating the critical view of the manuscript and acknowledge the identified importance of the topics addressed by the reviewer. The modifications of the manuscript following the recommendations of the reviewer have improved the manuscript and addressed the feedback given by the reviewer. All the changes are marked with track changes in the Manuscript file.*

 Point 1: There is very little engagement with the literature. This is at the beginning of the paper and is mainly concerned about communciation. However, the realities of scientific communication through social media have not been explored.

*Answer 1: We acknowledge the importance of engaging with the existing literature comprehensively, therefore we addressed this comment by rewriting the Discussion in order to be more engaged with the literature presented in the Introduction. In the Introduction section we provide findings of existing literature on the studied field. The identified gaps led us to create the main objective of our study. We connected the findings form the existing literature with the main findings of this study in the Discussion section.*

 Point 2: The structural characteristics of the 'networks' of the InnoRenew CoE on these three different channels, have been provided at the beginning of the Materials and methods section. This demographic data has not been used to evaluate the 'measures of engagement' on this different platforms.

*Answer 2: This was not the goal of our study, however we provided a description of the audience at the InnoRenew CoE social media channels, since we are aware that the characteristics of the audience is important for the engagement. We rewrote the Discussion section in order to enhance this aspect as a limitation of our study, and added the recommendation for future studies.*

 Point 3: The discussion is mainly based on the number of impressions, shares and comments. The differences between these 'measures of engagement' by different users has not been established in enough depth. For example, there is a claim that "users may not be comfortable commenting on topics connected to sustainable construction, or that they are not interested in engaging in conversations on social media to develop new relationships." As far as the paper is concerned, this correlation is now based on any evidence.

*Answer 3: We acknowledge that knowing the exact characteristics of the audience, the possible audience relapse and similar issues is a limitation of our study. We rewrote this part in the Discussion section to highlight it better. As for the quoted finding of our study regarding users not being comfortable to comment on topics on social media, this is the interoperation of the result which is also in line with previous studies that are cited in the manuscript, e.g.* *( Ellison et al., 2014* *)*
